# Advantages and pitfalls in utilizing artificial intelligence for crafting medical examinations: a medical education pilot study with GPT-4

**DOI:** 10.1186/s12909-023-04752-w

**Published:** 2023-10-17

**Authors:** Klang E, Portugez S, Gross R, Kassif Lerner R, Brenner A, Gilboa M, Ortal T, Ron S, Robinzon V, Meiri H, Segal G

**Affiliations:** 1 The Sami Sagol AI Hub, ARC Innovation Center, Chaim Sheba Medical Center. Affiliated to the Faculty of Medicine, Tel-Aviv University, Ramat Aviv, Israel; 2 Silesia Medical University, Katowice, Poland; 3 Division of Psychiatry, the Chaim Sheba Medical Center, Tel-Hashomer, Ramat Gan, Israel. Affiliated to the Faculty of Medicine, Tel-Aviv University, Ramat Aviv, Israel; 4 Department of Pediatric Intensive Care, The Edmond and Lily Safra Children’s’ Hospital, Chaim Sheba Medical Center. Affiliated to the Faculty of Medicine, Tel-Aviv University, Ramat Aviv, Israel; 5 Obstetrics and Gynecology Division, Chaim Sheba Medical Center. Affiliated to the Faculty of Medicine, Tel-Aviv University, Ramat Aviv, Israel; 6 Infection Prevention and Control Unit, Chaim Sheba Medical Center. Affiliated to the Faculty of Medicine, Tel-Aviv University, Ramat Aviv, Israel; 7 Education Authority, Chaim Sheba Medical Center. Affiliated to the Faculty of Medicine, Tel-Aviv University, Ramat Aviv, Israel; 8 Department of Surgery and Transplantation B, Chaim Sheba Medical Center. Affiliated to the Faculty of Medicine, Tel-Aviv University, Ramat Aviv, Israel

**Keywords:** Artificial intelligence, Chat GPT, Medical examinations, Multiple choice questions

## Abstract

**Background:**

The task of writing multiple choice question examinations for medical students is complex, timely and requires significant efforts from clinical staff and faculty. Applying artificial intelligence algorithms in this field of medical education may be advisable.

**Methods:**

During March to April 2023, we utilized GPT-4, an OpenAI application, to write a 210 multi choice questions-MCQs examination based on an existing exam template and thoroughly investigated the output by specialist physicians who were blinded to the source of the questions. Algorithm mistakes and inaccuracies, as identified by specialists were classified as stemming from age, gender or geographical insensitivities.

**Results:**

After inputting a detailed prompt, GPT-4 produced the test rapidly and effectively. Only 1 question (0.5%) was defined as false; 15% of questions necessitated revisions. Errors in the AI-generated questions included: the use of outdated or inaccurate terminology, age-sensitive inaccuracies, gender-sensitive inaccuracies, and geographically sensitive inaccuracies. Questions that were disqualified due to flawed methodology basis included elimination-based questions and questions that did not include elements of integrating knowledge with clinical reasoning.

**Conclusion:**

GPT-4 can be used as an adjunctive tool in creating multi-choice question medical examinations yet rigorous inspection by specialist physicians remains pivotal.

## Background

The global healthcare system faces a pressing challenge: increasing the number of healthcare professionals, particularly physicians, without impairing the quality of their education (1). Competency-based medical education (CBME) addresses this issue; however, written knowledge tests remain an essential component in evaluating the basic knowledge acquired by medical school graduates (2). As a result, the demand for creating multiple-choice questions (MCQs) for healthcare professionals' examinations, especially for medical students, is expected to rise, further complicating an already difficult task (3).

Each manager and leader in systems engaged with medical education worldwide, should ask themselves “who are my examination writers and what are their qualifications? What tests are employed to ensure that their product is of high quality, not only in terms of content but also in terms of their examinations’ methodology?” The fact that a healthcare professional is experienced and talented in his realm of practice does not indicate his ability and experience in writing MCQs (4). This realm of competencies encompasses a wide range of skills and expertise such as: syllabus tracking, topics’ selection, adhering to the correct rules of medical case presentation, directing the question to a certain domain (e.g., diagnosis or treatment) and rightfully choosing the way and extent of discrimination between the correct answers and false ones.

Since the realm of written tests in medicine is based on combining acquired knowledge and pre-determined methodology, it is only prudent to assume that in the future, the task of writing examinations in medicine would become another output of artificial intelligence applications (5,6). Recent advances in this technology have proven this assumption to be correct and reality is overriding imagination. The artificial intelligence application “GPT-4” developed by a private firm known as “Open-AI” – has successfully passed the USMLE, United States Medical Licensing Examination (7). It is only reasonable to hypothesize that this private firm application would be successful, no less, in writing such examinations – as modifications of existing ones and as de-novo MCQs writing.

Recognizing the potential benefits of applying artificial intelligence algorithms in exam writing, we describe the results of utilizing GPT-4 to write a 210 MCQs medical examination. The number of questions was chosen according to the number of questions included in our national medical licensing examination. We provide a qualitive description of the test, evaluated by various domain experts and present the challenges of this method.

## Methods

This study did not include research in humans. All methods were carried out in accordance with relevant guidelines and regulations. We have tested GPT-4 abilities in the realm of medical written, MCQs tests—in the challenges of creating new questions on the basis of existing ones (therefore, tracking the exam’s original syllabus) and maintaining high quality of both content and examination-constructing methodology. The GPT-4 model used did not go through any specific training in the fields / clinical disciplines included in the generated examination.

We inserted a request/prompt to GPT-4 asking it to rewrite a 210 MCQs test, and to create a new test based on the former one. The relevant, preliminary prompt given to the algorithm is shown in Table 1.

**Table 1:** Preliminary prompt

*Could you please generate Multiple Choice Questions (MCQs) on Internal Medicine for medical students? They should be of the same difficulty level as the examples I've provided. Please start numbering from 1, and label the choices from a-d, marking the correct answer with an asterisk?*	

Since the preliminary prompt resulted in short, informative questions, without clinical background, we reiterated our request by giving the algorithm another prompt, as presented in Table 2.

**Table 2:** Secondary prompt

*Could you please generate Multiple Choice Questions (MCQs) on Internal Medicine for medical students? They should be of the same difficulty level as the examples I've provided.* *Please start numbering from 1, and label the choices from a-d, marking the correct answer* *with an asterisk. Please write 5 as knowledge questions and 5 with "clinical history" questions*	

After receiving the algorithm initial output, we did a quality assurance phase by introducing the GPT-4 generated MCQs to five specialist physicians in the different test domains: internal medicine, general surgery, obstetrics and gynecology, psychiatry and pediatrics. The five specialists were blinded to the source of questions: they did not know the purpose of the study but only knew that they are serving as researchers in a quality-assuring study for MCQs writing in medicine. Also, their feedbacks were presented separately and they were blinded to each other’s opinions on the MCQs quality and professional level. They were also prompted to assess the correctness and appropriateness of the examination MCQs relating to previous versions of this test.

The researchers collected the specialists’ feedbacks and sorted them according to different potential errors: a). wrong questions (content mistakes included in the question stem), b). wrong answers (content mistakes included in one or more options, either intended to be correct or incorrect), c). methodological faults in either questions or answers (i.e., questions requiring eliminating options rather than choosing the correct ones), d). interdisciplinary mistakes (i.e., a mismatch between the question content and the intended focus of specific medical domain).

## Results

All data generated or analyzed during this study are included in this published article. We included all quantitative data regarding the number of questions written and number of rejects presented by the specialists. Also, we present in this article all qualitative data presenting all verbal rejects presented by specialists regarding the AI-generated MCQs. Overall, GPT-4 performance was extremely rapid and efficient. All questions were generated according to the secondary prompt and were introduced to five, blinded specialists who were not aware to the research question and to the optional writing of questions by an artificial intelligence algorithm. Of note, currently there is no option to write image-based questions, which is a major limitation in the field of medicine. In addition, the algorithm had difficulty differentiating between close disciplines, e.g., distinguishing between general surgery versus gynecological pathologies requiring surgical treatment in clinical scenarios addressing the lower abdomen.

Only one question (0.5%) out of 210 required replacements due to a completely mistaken answer. This question was in the domain of surgery. A total of 13 questions had more than one possible matching answer due to incomplete clinical information in the question stem, or optional answers that could not be definitively differentiated from each other. These questions and answers were not replaced but necessitated correction and re-writing in a better precision of the question stem or the answer options.

In addition, 3 questions presented patients’ age that was unconcordant with the clinical description (categorized above either as wrong questions or wrong answers). Such mistakes were included in disciplines that included questions that could be “age sensitive”: gynecology and pediatrics. For example, a question presenting a 38-year-old woman with irregular menses as postmenopausal. Also, one mistake that could be classified as “gender sensitive”, once again, in gynecology, when an abdominal complaint of a male was questioned, and the optional answers included ectopic pregnancy and ovarian cyst rupture.

In the chapter of internal medicine, two questions were considered, by specialist physicians, too easy and replaced, although considered qualifying.

In 2 cases, our specialists defined terminology used by GPT- 4 as being outdated or inaccurate: using the term SIRS (Systemic Inflammatory Response Syndrome) in the field of surgery and the term amenorrhea instead of irregular menses. Both questions necessitated correction but otherwise were judged as qualified.

Overall, 3 questions were identical / appeared twice in the same test and necessitated writing new ones. These questions were in the field of surgery. Additionally, three questions needed replacement as they presented a repeated topic that exceeded its appropriate weight within the exam syllabus. These questions were in the domain of internal medicine. Two questions were in the elimination format, which is considered methodologically flawed, despite the absence of such question types in the provided example questions.

One of the questions had wrong spelling, writing “GI track” instead of “GI tract”.

Overall, the majority of problematic questions were in the field of surgery, reaching up to 30% of questions in this subject. It is worth mentioning that this is the only subject in which the algorithm provided an incorrect answer. In the chapter of gynecology, 20% of the questions had inaccuracies, most commonly due to lack of relevant clinical descriptions. In pediatrics and internal medicine only 10% of the questions needed some kind of correction. All MCQs written by GPT-4 in psychiatry qualified and did not necessitate corrective measures. It should be emphasized that the original examination, serving as an example for GPT-4 had no such inaccuracies.

Presented hereafter are examples for questions necessitating replacement or correction:

- Example 01. A). A question necessitating change of the preliminary prompt due to lack of clinical case description at the base of the question.

### Which of the following is a negative symptom of schizophrenia?

A. Hallucinations
B. Delusions
C. Anhedonia
D. disorganized speech

- Example 01. B). A question generated according to the second prompt, relying on a clinical case presentation as a basis for the knowledge question.

### A 22-year-old male presents with disorganized speech, delusions, and hallucinations. These symptoms have been present for the past 3 months. What is the most likely diagnosis?

A *schizoaffective disorder*
B *Schizophrenia*
C *Major depressive disorder with psychotic features*
D *bipolar disorder with psychotic features*

- Example 02. A). A question presenting without a correct answer (content mistake).

### A 45-year-old woman presents with a painful, red, swollen left leg. Duplex ultrasound shows an occlusive thrombus in the left superficial femoral vein. What is the most appropriate initial treatment?

A. *Surgical thrombectomy*
B. *Anticoagulation therapy*
C. *Compression stockings*
D. *Elevation of the affected limb*

- Example 02. B). Corrected question (option b added manually as the correct answer).

### A 45-year-old woman presents with a painful, red, swollen left leg. Duplex ultrasound shows a small, non-occlusive thrombus in the left superficial femoral vein. What is the most appropriate initial treatment?

A. *Surgical thrombectomy*
B. *Non-Steroidal Anti-Inflammatory Drug*
C. *Compression stockings*
D. *Elevation of the affected limb*

- Example 02 shows a question with incorrect answer regarding the treatment of superficial vein thrombosis. The answer provided by the algorithm was anticoagulation therapy, which is the correct answer for deep vein thrombosis. The above question describes a patient with superficial vein thrombosis, which is treated using non-steroidal anti-inflammatory drugs.
- Example 03. A). A question using out-of-date terminology.

### A 71-year-old woman is hospitalized with an Inflammatory Bowel Disease exacerbation. During her hospitalization she has fever with leukocytosis and is diagnosed with SIRS -Systemic Inflammatory Response Syndrome. Which of the following statements is correct regarding the pathophysiology of her condition?

A. *It is activated mainly by the Innate Immune system*
B. *Vasoconstriction is caused mostly by Histamine and Bradykinin*
C. *Anti-cytokine medications such as Anti-TNF were proven effective in decreasing mortality*
D. *The Cardiac Index is decreased due to an increase in the Afterload*

- Example 03 shows a question using the term SIRS, which is no longer used as part of sepsis diagnostic criteria that were replaced two years ago.
- Example 04. A). A question presenting with an inaccurate terminology.

### A 34-year-old woman presents with amenorrhea for three months. She has a history of irregular menstrual cycles and hirsutism. Her BMI is 32. What is the most likely diagnosis?

A. *Premature ovarian failure*
B. *Polycystic ovary syndrome (PCOS)*
C. *Hyperprolactinemia*
D. *Hypothyroidism*

- Example 04. B). A correction of the question, replacing the incorrect professional term.

### A 34-year-old woman presents with irregular menses (last was three months ago). She has a history of irregular menstrual cycles and hirsutism. Her BMI is 32. What is the most likely diagnosis?

A. *Premature ovarian failure*
B. *Polycystic ovary syndrome (PCOS)*
C. *Hyperprolactinemia*
D. *Hypothyroidism*

- Example 04 shows a question mistakenly using the term amenorrhea when the correct description had to be irregular menses.
- Example 05. A). Presenting 38-year-old woman as postmenopausal (age sensitive question).

### A 38-year-old woman presents with postmenopausal bleeding. Endometrial biopsy reveals atypical endometrial hyperplasia. What is the most appropriate management for this patient?

A. *Progesterone therapy*
B. *Hysterectomy*
C. *Endometrial ablation*
D. *Observation and repeat biopsy in 3 months*

- Example 05. B). A question correction, replacing the age of the patient to 58 years old woman.

### A 58-year-old woman presents with postmenopausal bleeding. Endometrial biopsy reveals atypical endometrial hyperplasia. What is the most appropriate management for this patient?

A. *Progesterone therapy*
B. *Hysterectomy*
C. *Endometrial ablation*
D. *Observation and repeat biopsy in 3 months*

- Example 06. A). A question presenting a “pediatric patient” without specifying the fact that he should be an infant (age-sensitive question).

### A pediatric patient presents with a "blueberry muffin" rash, hepatosplenomegaly, and jaundice. Which of the following is the most likely cause?

A. *Cytomegalovirus (CMV) infection*
B. *Congenital rubella infection*
C. *Congenital syphilis*
D. Congenital toxoplasmosis

- Example 06. B). The age of the patient was added for the appropriateness of optional answers.

### A 4-month-old infant presents with a "blueberry muffin" rash, hepatosplenomegaly, and jaundice. Which of the following is the most likely cause?

A. *Cytomegalovirus (CMV) infection*
B. *Congenital rubella infection*
C. *Congenital syphilis*
D. *Congenital toxoplasmosis*

- Example 07. A question deemed as wrong, necessitating replacement.

### Which of the following pediatric conditions is characterized by recurrent episodes of paroxysmal vertigo, tinnitus, and hearing loss?

A. *Meniere's disease*
B. *Benign paroxysmal positional vertigo*
C. *Migraine-associated vertigo*
D. *Acoustic neuroma*

- Example 07 shows a question in which the algorithm associated Meniere’s disease, a disease classically appearing between the age of 20 to 60, as a viable option (intended to be the correct answer) in children. This mistake should be classified as age-sensitive.
- Example 08. A). A question presenting a male patient, with two of the provided answers describing gynecological pathologies (gender sensitive mistake).

### A 56-year-old male presents with acute onset of severe left lower quadrant pain, fever, and nausea. Upon examination, there is tenderness and guarding in the left lower quadrant. What is the most likely diagnosis?

A. *Acute appendicitis*
B. *Diverticulitis*
C. *Ovarian cyst rupture*
D. *Ectopic pregnancy*

- Example 08. B). The gynecological pathologies were replaced.

### A 56-year-old male presents with acute onset of severe left lower quadrant pain, fever, and nausea. Upon examination, there is tenderness and guarding in the left lower quadrant. What is the most likely diagnosis?

A. *Acute appendicitis*
B. *Diverticulitis*
C. *Acute cholecystitis*
D. *Liver abscess*

- Example 09. A question presenting a clinical case of Lyme disease, without mentioning traveling to high incident countries. A mistake potentially classified as geographically sensitive.

### A child presents with a "bull's-eye" rash, fever, and joint pain. Which of the following is the most likely diagnosis?

A. *Rocky Mountain spotted fever*
B. *Lyme disease*
C. *Erythema multiforme*
D. *Stevens-Johnson syndrome*

- Example 09 shows an example of a question aiming for a disease that has specific geographical distribution, without mentioning traveling to endemic areas.
- Example 10. A). A question lacked information, resulting in the incorrectness of the marked answer. While the question asks about the most common cause of anemia in children, the marked answer was physiological anemia, which is true for neonates but not for children. Once again, an age-sensitive question.

### Which of the following is the most common cause of anemia in children?

A. *Iron deficiency*
B. *Sickle cell disease*
C. *Thalassemia*
D. *Physiologic anemia*

- Example 10. B). The question was changed to “neonates” instead of “children” to match the correct answer.

### Which of the following is the most common cause of anemia in neonates?

A. *Iron deficiency*
B. *Sickle cell disease*
C. *Thalassemia*
D. *Physiologic anemia*

- Example 11. An elimination request in a too-short question (methodological mistake).

### Which of the following is NOT a core symptom of autism spectrum disorder (ASD)?

A. *Deficits in social communication*
B. *Restricted, repetitive patterns of behavior*
C. *Sensory sensitivities*
D. *Excessive worry and anxiety*

- Example 12. Two questions showing the tendency of the algorithm to form too simplistic questions without need for clinical integration.

### Which of the following is the primary neurotransmitter implicated in the pathophysiology of schizophrenia?

A. *Serotonin*
B. *Norepinephrine*
C. *Dopamine*
D. *GABA*

### What is the most common cause of bronchiolitis in infants and young children?

A. *Respiratory syncytial virus (RSV)*
B. *Influenza virus*
C. *Parainfluenza virus*
D. *Adenovirus*

## Discussion

In this study we aimed to evaluate the performance of GPT-4, an artificial intelligence application, in generating multiple-choice questions (MCQs) for medical exams. Overall, when compared to the tedious process of gathering examination writers from five clinical disciplines, the performance of GPT-4 was rapid and efficient, with the majority of questions deemed suitable for the exam by a panel of specialists, blinded to the source of the questions. However, some errors were identified, including mistaken answers, age and gender inconsistencies, repeated questions, and methodological flaws.

The healthcare system worldwide faces a critical dilemma: the need to enhance the quantity of healthcare professionals, particularly physicians, while ensuring the excellence of their education (8). Since written knowledge tests continue to play a vital role in assessing the core knowledge acquired by medical school graduates, the task of generating multiple-choice questions (MCQs) for healthcare professionals' examinations, becomes even more challenging due to the anticipated increase in demand. As written by Alexander Pope (9), more than 200 years ago, “to err is human” and indeed, expertise in a healthcare profession does not automatically translate to the ability to write effective multiple-choice questions (MCQs), which requires a separate set of capabilities. As a result, there is a constant need to reflect on the qualifications of examination writers and the methods used to ensure the quality of written exams.

Recent advances in the subject of artificial intelligence (AI), specifically GPT-4 which is a large language model, offers valuable contributions in the field of education (10,11). It can be utilized for automated scoring of student papers, easing the burden of grading for teachers (11,12). In addition, it can be used as a teacher assistant, providing help in exercises and quizzes generation for both practice and assessment (13,14). Additionally, GPT-4 can enhance personalized study plans, further contributing to student understanding and facilitating tailored learning experiences based on individual needs and preferences. GPT-4 was the first algorithm to successfully pass the United States Medical Licensing Examination (USMLE), reaching a very high score of 87 (15). Lastly, it was also demonstrated that it was capable of generating USMLE-like exam questions that are challenging to distinguish from human-generated questions, showing the potential of GPT-4 to assist in exam preparation (16).

The objective of our study was to propose the utilization of GPT-4 as an aid in exam preparation while identifying its limitations, helping reduce the workload of the physician-educator (17). In general, the process of generating tests with GPT-4 was fast and efficient in terms of time. However, approximately 15% of the questions generated from the detailed prompt required some correction, primarily due to inaccuracies in content or faulty methodology.

The main revised questions in terms of wrong content basis were due to the lack of sensitivity in specific topics, ignoring the requirements to use specific age, gender or geographical location for either the question or the answers. Such lack of sensitivity was mentioned in a recent paper, describing a user getting the wrong answer generated by GPT-4 in chemistry, providing an answer with wrong units (18). This limitation seems to be due to GPT-4’s limitations in reasoning recalled knowledge (19).

One of GPT-4’s major limitations is that its primary source of training data is the internet, which can be inaccurate and unreliable (18,20). This results in the necessity to validate GPT-4 output for tasks requiring high levels of credibility (20). A less concerning example is typos, e.g., “GI track” instead of “GI tract”. While more concerning inaccuracies can result in providing incorrect answers, e.g., treating superficial vein thrombosis using anticoagulation instead of NSAIDs, due to either inaccurate training data or “factualness error” which is caused by lack of training data in the specific, asked subject (19).

Our findings of GPT-4 limited integration of knowledge and clinical reasoning, align with recent papers highlighting the challenge of logical reasoning (20), incapability of GPT-4 to generate novel findings based on existing knowledge and limited capacity to innovate (21). This is the result of GPT-4 architecture, which focuses on providing coherent responses from its vast knowledge base rather than extrapolating new insights or hypothesizing connections through identification of hidden patterns and relationships (18).

## Conclusions

Medical, MCQs-based examination writing by GPT-4 is feasible yet necessitates rigorous inspection by specialist physicians. Learning the characteristic flaws and mishaps of GPT-4 in this task is essential for faculty members intending to apply AI potential in this realm. We did not challenge artificial intelligence with questions containing figures, tables of graphs that are sometimes desirable.

### Limitations

This study concentrated on the production of one, multidisciplinary MCQ examination. Also, we describe our experience with GPT-4 which will be updated in the future. Our findings lay the fundaments for further, similar studies.

## Data Availability

All data and materials that are not included in the manuscript are available upon request from the corresponding author.
